# Successful use of continuous subcutaneous Foslevodopa/Foscarbidopa in Parkinson's disease with bipolar disorder without psychiatric destabilization

**DOI:** 10.1016/j.prdoa.2026.100475

**Published:** 2026-06-23

**Authors:** Joanna Siuda, Justyna Gawryluk, Amelia Krupczak, Mateusz Toś

**Affiliations:** a Department of Neurology, Faculty of Medical Sciences in Katowice, Medical University of Silesia, Katowice, Poland; b Students' Scientific Association, Department of Neurology, Faculty of Medical Sciences in Katowice, Medical University of Silesia in Katowice, Poland

**Keywords:** Foslevodopa/foscarbidopa, Advanced Parkinson’s disease, Bipolar disorder, Device-aided therapy, Mood stability

## Abstract

We report a patient with advanced Parkinson's disease and treatment-resistant bipolar disorder in whom continuous subcutaneous foslevodopa/foscarbidopa infusion improved motor fluctuations without psychiatric destabilization despite prior dopaminergic sensitivity. This case suggests that LDp/CDp may be a therapeutic option even in patients with bipolar disorder.

Effective treatment of bipolar disorder (BD) may represent a significant clinical challenge in people with Parkinson's disease (PwP), due to the potentially negative impact of psychiatric treatment on motor symptoms, as well as the risk of mental state destabilization during intensification of dopaminergic therapy [1]. Consequently, the coexistence of BD and PD may substantially limit the choice of appropriate antiparkinsonian medications and the optimization of treatment, including the use of advanced therapies.

Continuous subcutaneous infusion of foslevodopa/foscarbidopa (LDp/CDp) is one of the newest device-aided therapy (DAT) options for advanced Parkinson's disease, providing stable dopaminergic stimulation and effective reduction of motor fluctuations [2], [3]. However, recent observations suggest that its use may be limited by adverse psychiatric effects [4], [5], [6].

We present the case of a patient with treatment-resistant BD in whom LDp/CDp therapy was successfully implemented without psychiatric deterioration.

A 47-year-old male with BD diagnosed at 18 developed a resting tremor of the right upper limb at 36, followed by progressive rigidity, bradykinesia, and gait impairment. Multiple antipsychotics and mood stabilizers were previously used, often resulting in worsening of motor symptoms (Fig. 1). Drug-induced parkinsonism was initially suspected, and propranolol was introduced without clinical benefit. Due to further progression of motor symptoms and the need to control psychiatric manifestations, clozapine was initiated but was discontinued due to excessive sedation and elevated liver enzymes. Stabilization of BD was ultimately achieved with lurasidone, quetiapine, and lamotrigine, while biperiden improved tremor. The patient had also remained in ongoing psychodynamic psychotherapy since 2019. A DaTSCAN SPECT study demonstrated reduced presynaptic dopaminergic tracer uptake in the striatum, predominantly affecting the left putamen, supporting the diagnosis of PD. Levodopa therapy was initiated and gradually escalated, resulting in initial motor improvement. Over time, disease progression led to motor fluctuations, including unpredictable wearing-off, delayed ON, nocturnal rigidity, and morning akinesia, along with non-troublesome peak-dose dyskinesias. An attempt to increase the levodopa dose to 1500 mg/day resulted in troublesome dyskinesias and the occurrence of a hypomanic episode, requiring psychiatric intervention and reduction of the levodopa dose. This significantly limited further optimization of dopaminergic therapy. A trial of entacapone did not provide meaningful benefit. Given the risk of psychiatric destabilization, dopamine agonists, MAO-B inhibitors, and amantadine were not introduced.

**Fig. 1:**
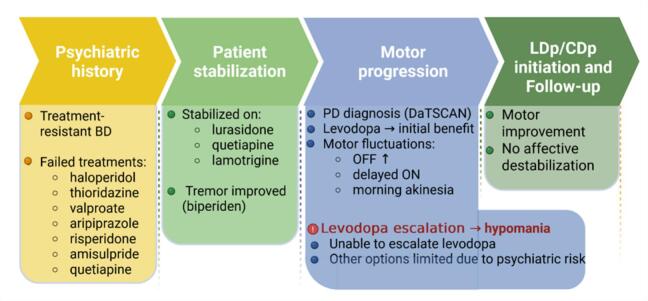
Clinical course and treatment timeline in a patient with Parkinson's disease and treatment-resistant bipolar disorder. BD, bipolar disorder; PD, Parkinson’s disease; LDp/CDp, foslevodopa/foscarbidopa.

Due to disabling motor fluctuations and lack of further oral treatment options, LDp/CDp therapy was initiated. Deep brain stimulation (DBS) and continuous subcutaneous apomorphine infusion (CSAI) were not considered due to psychiatric risk, and the patient declined levodopa/carbidopa intestinal gel therapy (LCIG). Treatment was started at a daytime infusion rate of 0.40 mL/h, calculated according to the Summary of Product Characteristics, while the nighttime rate was conservatively set at 0.15 mL/h, because of concerns regarding psychiatric tolerability. Escalation of the nighttime dose improved morning akinesia but resulted in reduced sleep duration and the emergence of restlessness. Given the risk of BD exacerbation, the nighttime infusion was discontinued on the third day of hospitalization, and estazolam was introduced following psychiatric consultation, resulting in normalization of sleep. The patient was discharged on daytime infusion only.

In the subsequent months, estazolam was gradually discontinued without recurrence of sleep disturbances. At 10-month follow-up, the patient remained psychiatrically stable under treatment with lurasidone, quetiapine, and lamotrigine. At the same time, significant motor improvement was observed, including an approximately 70% reduction in OFF time and absence of troublesome dyskinesias.

This case illustrates the challenge of managing advanced PD in the context of coexisting BD, where treatment decisions are constrained by the risk of psychiatric destabilization. In our patient, levodopa dose escalation led to a hypomanic episode, significantly limiting further optimization of dopaminergic therapy.

Although psychiatric adverse effects have been reported with LDp/CDp therapy, it was well tolerated in this case, without evidence of affective destabilization. This is particularly relevant given the patient's history of treatment-resistant BD and previous sensitivity to dopaminergic dose escalation. Available reports suggest that psychiatric adverse effects during LDp/CDp therapy tend to occur in clinically vulnerable patients, particularly those with cognitive impairment, previous psychotic or confusional episodes, and potentially modifiable medication-related risk factors such as concomitant catechol-*O*-methyltransferase inhibitor (COMT-I) use [4], [5], [6]. In some cases, these adverse effects required dose reduction, antipsychotic treatment, or discontinuation of LDp/CDp. Our patient differed from these previously reported cases in that the main psychiatric risk factor was treatment-resistant BD, rather than cognitive impairment or a history of psychotic symptoms. These findings suggest that, with careful dose titration and close neuropsychiatric collaboration, LDp/CDp may be considered in selected patients with pre-existing mood disorders. However, even a relatively conservative nighttime LDp/CDp infusion rate in our patient (0.15 mL/h; approximately 38% of the daytime rate) was associated with restlessness and reduced sleep duration, likely reflecting psychiatric activation rather than a primary sleep-related effect. This observation underscores the need for caution when introducing nocturnal dopaminergic stimulation in patients with underlying mood disorders.

This case suggests that LDp/CDp may represent a therapeutic option in selected patients with advanced PD and coexisting BD, in whom treatment choices are limited by psychiatric comorbidity, provided that close psychiatric monitoring is ensured.

## Ethical approval and patient consent

No institutional review board approval was required for this study. Written informed consent was obtained from the patient for publication of the case.

## CRediT authorship contribution statement

**Joanna Siuda:** Writing – review & editing, Supervision, Project administration, Investigation, Data curation, Conceptualization. **Justyna Gawryluk:** Writing – review & editing, Investigation, Data curation. **Amelia Krupczak:** Writing – original draft, Investigation. **Mateusz Toś:** Writing – original draft, Software, Project administration, Investigation, Data curation, Conceptualization.

## Declaration of competing interest

The authors declare the following financial interests/personal relationships which may be considered as potential competing interests: Mateusz Tos reports a relationship with AbbVie Inc that includes: travel reimbursement. Justyna Gawryluk reports a relationship with AbbVie Inc that includes: speaking and lecture fees and travel reimbursement. Joanna Siuda reports a relationship with AbbVie Inc that includes: consulting or advisory, speaking and lecture fees, and travel reimbursement. Joanna Siuda reports a relationship with EVER Pharma Holding that includes: speaking and lecture fees and travel reimbursement. Joanna Siuda reports a relationship with QPharma Inc that includes: speaking and lecture fees. Joanna Siuda reports a relationship with Eli Lilly that includes: consulting or advisory. If there are other authors, they declare that they have no known competing financial interests or personal relationships that could have appeared to influence the work reported in this paper.
